# Comparison of Necrotizing Enterocolitis in Pre-mature Infants vs. Term-Born Infants With Congenital Heart Disease

**DOI:** 10.3389/fped.2021.802607

**Published:** 2021-12-20

**Authors:** Gabriela Frid, Marina Reppucci, Tony Lum, Megan Paul, Howard Seiden, Brian A. Coakley

**Affiliations:** ^1^The Icahn School of Medicine at Mount Sinai, New York, NY, United States; ^2^Department of Surgery, The Icahn School of Medicine at Mount Sinai, New York, NY, United States; ^3^Division of Pediatric Cardiology, Department of Pediatrics, The Icahn School of Medicine at Mount Sinai, New York, NY, United States; ^4^Division of Pediatric Surgery, Department of Surgery, The Icahn School of Medicine of Mount Sinai, New York, NY, United States

**Keywords:** necrotizing enterocolitis (NEC), prematurity, congenital heart disease, laparotomy, ischemic bowel

## Abstract

**Purpose:** Necrotizing enterocolitis (NEC) is a serious illness that occurs among premature infants and term-born infants with congenital heart disease (CHD). Prior studies have suggested these two groups may experience different disease entities. We sought to evaluate if there are differences in disease characteristics between these two populations.

**Materials and Methods:** A retrospective chart review of infants treated for Bells stage 2-3 NEC from 2011 to 2020 was performed. Demographic information, CHD diagnoses and clinical data were recorded. Prior to data analysis, patients were divided into two groups: term-born patients with CHD (TC) and premature patients without CHD (PT).

**Results:** 99 patients were analyzed−23 TC patients and 76 PT patients. Platelet counts (222.7 ± 176.1 vs. 310.2 ± 174.5 cells/uL, *P* = 0.03) and C-reactive protein (CRP) levels (53.6 ± 81.7 vs. 117.6 ± 90.4 mg/L, *P* < 0.001) were significantly higher among the PT group. In addition, PT patients were more likely to develop pneumatosis (30.4 vs. 68.4%, *P* = 0.002) than TC patients. NEC-specific mortality was similar between both groups of patients.

**Conclusions:** When compared to TC patients, PT patients had higher CRP levels, higher platelet counts and more commonly developed pneumatosis. These factors may point toward a difference in disease pathophysiology regarding NEC development in premature patients vs. term-born patients with CHD.

## Introduction

Necrotizing enterocolitis (NEC) is an inflammatory gastrointestinal disease among infants, which confers a mortality risk of 25–33% (1–3). Numerous risk factors have been pinpointed regarding the development of NEC, with prematurity and low birth weight being the most consistently identified (4). While NEC is primarily a disease of premature infants, 10% of infants diagnosed with NEC are born at full term (5). Among term-born infants with NEC, congenital heart disease (CHD) has previously been demonstrated to be a predisposing factor (6, 7). Given that patients with CHD frequently do not possess the common risk factors of prematurity or low birth weight, it remains possible that the development and characteristics of NEC in preterm infants may differ from that of term-born infants with CHD. In addition, many patients with CHD have unique physiology which might affect their capacity for splanchnic oxygenation, such as systemically low levels of oxygen saturation, diminished cardiac output or “steal syndrome” resulting decreased visceral perfusion due to reversal of aortic flow during diastole (8). Further, although CHD has been identified as a risk factor for NEC in term-born infants, the risk of death among these patients, as compared to their preterm counterparts, remains unclear, with conflicting studies showing either improved or worse outcomes (6, 7). Prior studies have evaluated the differences in disease, and have suggested that although they present similarly, NEC may be a manifestation of two separate disease entities in these two different populations. The intent of this study was to assess clinical characteristics and outcomes to determine if NEC occurs via different pathophysiologic mechanisms in preterm patients as compared to term-born infants with CHD.

## Materials and Methods

A retrospective review of a prospectively-collected single-institution database including all patients diagnosed with NEC in the Neonatal Intensive Care Units (NICUs) of The Mount Sinai Health System from 2010 to 2021 was conducted. For inclusion in the database, patients were diagnosed with NEC by a board-certified neonatologist, board-certified pediatric surgeon or both. Any patients with Bell's stage 1 disease were excluded, as the diagnosis of NEC cannot be certain in such patients. All Bell's stage 2–3 patients were then categorized into two groups: premature patients without CHD (PT) or term-born congenital heart disease patients (TC). Prematurity was defined as birth prior to 37 weeks gestation. All cases of spontaneous intestinal perforation, defined as the presence of pneumoperitoneum without preceding pneumatosis in infants <1.5 kg, were excluded.

Anatomical and hemodynamic characterization of each patient's CHD was performed by a treating pediatric cardiologist based on echocardiographic data and the in-hospital clinical course. We analyzed various patient characteristics that may contribute to the development of NEC, such as gender, gestational age, birth weight, Apgar scores at 1 and 5 min, presence of patent ductus arteriosus (PDA) at the time of NEC onset, use of indomethacin, type of feeds initiated, timing of feeds initiation, maternal age, and pregnancy type (singleton vs. multiple). For all CHD patients, the type and status of their CHD was recorded. CHD patients were also assessed for the presence of steal syndrome, as defined by the reversal of diastolic flow in the descending aorta on echocardiography. Demographic data, Bell's stage, biochemical measures of acute illness and radiographic findings were recorded for all patients. For imaging data, both reports and actual images were reviewed by a faculty pediatric radiologist. Treatment strategies and clinical outcomes were also assessed. Sites of disease was recorded for all patients undergoing laparotomy.

Statistical analysis was performed via R statistical software. Categorical variables were assessed with either χ^2^ or Fisher's exact tests, while continuous variables were compared with Student's *t*-tests and single-factor ANOVA tests. This project was approved by the Institutional Review Board of The Mount Sinai Health System.

## Results

Altogether, 99 patients treated for NEC met criteria for inclusion−76 PT patients and 23 TC patients. Patient demographics and measures of acute illness are listed in Table 1. Racial and gender distributions were similar between the two groups. TC patients had a higher gestational age at birth (*P* < 0.001) and birthweight (*P* < 0.001) than PT patients. One- (*P* = 0.002) and 5-min (*P* = 0.015) APGAR scores were significantly higher in TC patients than in PT group. There was no difference in maternal age at conception, time to initiation of enteral feeds, time to diagnosis of NEC or utilization of human breast milk or formula across the two groups.

**Table 1:** Patient characteristics.

	**TC pts** **(***N*** = 23)**	**PT pts** **(*N* = 76)**	***P***
Maternal age at conception, years (mean ± stdev)	31.8 ± 5.1	32.0 ± 7.2	0.97
Gestational age at birth, weeks (mean ± stdev)	38.0 ± 2.0	28.2 ± 3.2	<0.001
**Gender**			
Female	11	32	0.64
Male	12	44	
**Ethnicity**			
White	3	14	0.82
Black	4	21	
Latino	5	8	
Other	3	22	
Unknown	8	11	
Birth weight, Kg (mean ± stdev)	2.9 ± 0.5	1.1 ± 0.3	<0.001
1-min APGAR (mean ± stdev)	8.0 ± 1.7	6.2 ± 2.8	0.01
5-min APGAR (mean ± stdev)	8.7 ± 0.6	7.6 ± 2.0	0.02
Time to initiation of feeds, days (mean ± stdev)	4.1 ± 4.7	5.9 ± 11.1	0.35
Initial feeding substrate employed			1
Human milk	18 (78.3)	59 (77.6)	
Formula	5 (21.7)	17 (22.4)	
Time to diagnosis, days (median ± IQR)	19 ± 14.5	17 ± 16	0.23
Weight at diagnosis, Kg (mean ± stdev)	3.5 ± 1.2	1.3 ± 0.4	<0.001
WBC at diagnosis, cells/uL (mean ± stdev)	15.6 ± 8.0	18.0 ± 15.6	0.57
Hemoglobin at diagnosis, gm/dL (mean ± stdev)	12.8 ± 1.8	11.2 ± 2.4	0.01
Platelet count at diagnosis, cells/uL (mean ± stdev)	222.7 ± 176.1	310.2 ± 174.5	0.03
C-reactive protein at diagnosis, mg/L (mean ± stdev)	53.6 ± 81.7	117.6 ± 90.4	<0.001
pH at diagnosis, (mean ± stdev)	7.4 ± 0.14	7.30 ± 0.19	0.22
Lactate at diagnosis, mmol/L (mean ± stdev)	2.9 ± 2.6	3.6 ± 3.9	0.68
Pneumatosis, (%)	7 (30.4)	52 (68.4)	0.002
Portal venous gas, (%)	4 (17.3)	15 (19.7)	1
Pneumoperitoneum, (%)	5 (21.7)	16 (21.1)	1
Bell's stage, (%)			1
Stage II	11 (47.8)	37 (48.7)	
Stage III	12 (52.2)	39 (51.3)	

The forms of congenital heart disease for patients in the TC group are shown in Table 2. The overwhelming majority of patients had either pulmonary atresia/stenosis with or without a ventricular septal defect (VSD) (*N* = 10, 43.5%) or some form of left-sided obstructive heart disease (*N* = 9, 39.1%). Similar percentages of both the TC (10/23, 43.5%) and the PT (34/76, 44.7%) had a PDA at the time of diagnosis. While 13 of the TC patients did not have a PDA, eight of them did have a surgical shunt between the systemic and pulmonary circulations. The vast majority (21/23, 91.3%) of these infants suffered from low oxygen levels of blood circulated to the lower body, while slightly more than half (14/23, 60.9%) had diminished perfusion to the lower body.

**Table 2:** Types of congenital heart disease.

**Classification of CHD**	**PDA or shunt**	**DDSC**	**DDPC**	**A**	**B**	***N*** **= 23**
**Pulmonary atresia/stenosis ± VSD**						10
Tricuspid/pulmonary atresia + RV hypoplasia	+	–	+	+	+	
Unbalanced AV canal + pulmonary atresia	–	–	+	–	+	
Pulmonary atresia + IVS	+	+	–	+	+	
Dysplastic pulmonary valve with severe PS	+	–	–	–	+	
Pulmonary atresia with IVS + hypoplastic TV/RV	+	–	+	+	+	
Tricuspid/pulmonary atresia + DORV	+	–	+	+	+	
Tricuspid/pulmonary atresia + DORV	+	–	+	+	+	
Tricuspid/pulmonary atresia + DORV	+	–	+	+	+	
Tetralogy of fallot + unbalanced AV canal	+	–	+	+	+	
Tricuspid atresia + NRGA	+	–	+	–	+	
**Center-sided obstructive heart disease**						9
Hypoplastic center heart syndrome	+	+	–	+	+	
Hypoplastic center heart syndrome	+	+	–	+	+	
Hypoplastic center heart syndrome	–	+	–	–	+	
Hypoplastic center heart syndrome	+	+	–	+	+	
Type B interrupted aortic arch	+	+	–	+	+	
Aortic coarctation	+	+	–	–	+	
Aortic coarctation	–	–	–	–	–	
Aortic coarctation	+	+	–	+	+	
Aortic coarctation	+	+	–	+	+	
**Isolated VSDs**						2
Multiple VSDs	–	–	–	–	–	
Single VSD	+	–	–	–	+	
Patent truncus arteriosus + severe vavlular regurgitation	–	–	–	–	+	1
Transposition of the great arteries + pulmonary atresia	+	–	+	+	+	1

*CHD, congenital heart disease; PDA, patent ductus arteriosus; VSD, ventricular septal defect; RV, right ventricle; AV, atriorventricular; IVS, interventricular septum; DORV, double-outlet right ventricle; NRGA, normally-related great arteries; A, diminished perfusion to the lower body; B, decreased systemic oxygen levels*.

At the time of diagnosis of NEC, mean levels for WBC, pH and lactate were not statistically different between the two groups (see Table 1). Hemoglobin levels were significantly higher in TC patients (*P* = 0.01) than they were for PT patients. Conversely, C-reactive protein (CRP) levels were significantly higher in the PT group than they were in the TC group (117.6 mg/L vs. 53.6 mg/L, *P* < 0.001). PT patients also displayed higher platelet counts at the time of NEC diagnosis than their TC counterparts (*P* = 0.03). The incidence of portal venous gas and pneumoperitoneum was not different between the two groups, while pneumatosis was significantly more common in the PT population (*P* = 0.002). Inotropic support was utilized with similar frequency across both groups.

Treatment-related outcomes are listed in Table 3. Laparotomy was employed with equal frequency among both groups, while peritoneal drainage was more often employed in the PT group (22.4 vs. 4.4%). At laparotomy, all three TC patients were found to have extensive disease of the colon. Conversely, only 45.5% (5/11) of the PT patients had colonic involvement of their disease. Among the 5 PT patients who had colonic involvement, 4 of them had involvement of the cecum and ascending colon only, while only 1 had disease distal to the ascending colon. Further, 45.5% (5/11) of the surgical PT patients had pan-intestinal ischemia, while none of the TC patients had such disease distribution (see Table 4).

**Table 3:** Treatment-related outcomes.

	**TC pts** **(***N*** = 23)**	**PT pts** **(***N*** = 76)**	***P***
Inotropic support required, (%)	13 (56.5)	30 (39.5)	0.23
Duration of antibiotic treatment, days (mean ± stdev)	15.4 ± 23.7	11.5 ± 6.4	0.15
**Surgical intervention required, %**			
Total	4 (17.4)	28 (36.8)	0.40
Peritoneal drainage	1 (4.4)	17 (22.4)	
Laparotomy	3 (13.0)	11 (14.5)	
Recurrent NEC, (%)	2 (8.7)	9 (11.8)	1
Weight at discharge, Kg (mean ± stdev)	4.4 ± 1.5	2.9 ± 1.1	<0.001
Length of stay, days (mean ± stdev)	96.5 ± 61.7	87.2 ± 57.9	0.61
Time from diagnosis to discharge, days (mean ± stdev)	68.3 ± 50.1	64.6 ± 56.0	0.83

**Table 4:** Anatomical distribution of disease.

	**TC** **(***N*** = 3)**	**PT** **(***N*** = 11)**
Small intestinal involvement	1	11
Pan-intestinal involvement	0	5
Colonic involvement	3	5
Right colon involvement	2	4
Disease distal to hepatic flexure	3	1

Post-NEC treatment complications were somewhat more common in PT patients than in TC patients (*P* = 0.06). However, no specific complication was more common among either group. Of the 18 PT patients who underwent peritoneal drainage, 8 died, 3 soon underwent laparotomy and 7 recovered from NEC without further surgical intervention. Recurrent NEC was equally common among the three groups (*P* = 0.4). The risk of NEC-specific mortality was fairly similar between the two groups, with between 13.0 and 23.7% of patients in each group succumbing to their illness. All of the TC patients who died had either a PDA or surgical shunt at time of death, while only 50% of the patients in the PT group who died had a PDA at the time of death.

## Discussion

This study revealed that while preterm infants without CHD who develop NEC and term-born infants with CHD who develop NEC are two very different patient populations, many markers of acute illness don't differ substantially between them. In fact, our findings revealed that multiple biochemical measures (white blood count, pH, lactate), radiologic findings (portal venous gas, pneumoperitoneum) and assessments of clinical severity (Bell's stage) were not different between these two groups of patients. Some of the noted differences, such as lower APGAR scores, lower hemoglobin levels and lower weights at diagnosis among the PT patients, seem attributable to the difference in gestational age between the two study groups. However, we also observed higher platelet counts (310.2 vs. 222.7 cell/uL, *P* = 0.03) and higher CRP levels (117.6 vs. 53.6 mg/L, *P* < 0.001) among the PT group. The finding of higher CRP levels among preterm infants with NEC has been reported by at least one other study (9). Given that CRP and platelets are known to be an acute phase reactants, this supports the presence of a more robust inflammatory response among PT patients than in TC patients. Further, this also suggests that PT patients may sustain an inflammatory-based disease, whereas TC patients are potentially more likely to suffer from an ischemic-based event. In fact, others have shown that the colon can be particularly susceptible to ischemic injury through a variety of mechanisms, including volume depletion, sepsis, mesenteric vasoconstriction and repercussion injury (10–13).

Interestingly, the prevalence of pneumatosis intestinalis was significantly higher in the PT population than in their TC counterparts (68.4 vs. 30.4%, *p* = 0.002). Others have speculated that pneumatosis intestinalis develops from an initial inflammatory insult, which then causes breakdown of mucosal barriers and allows for transmission of gas-forming bacteria into the wall of the intestine (14). It remains conceivable that the intestinal microbiome of preterm infants has not fully matured, leaving this brittle population particularly susceptible to the development of pneumatosis intestinalis as compared to term-born infants. To that end, several prior studies have shown alterations of the intestinal microbiome among premature infants (15–17).

Clinical outcomes were fairly similar between the the PT and TC groups. The risk of intestinal perforation was virtually identical (21.7% for TC patients and 21.1% for PT patients) for both groups and laparotomy was performed with similar frequency (13.0% in TC patients vs. 14.5% in PT patients) for both populations. Although not statistically significant, peritoneal drainage was employed more frequently in PT patients (22.4%) than in the TC group (4.4%). This likely reflects a practice pattern at our institution where patients are approached differently based on their gestational age and weight at the time of diagnosis, which is similar to the strategy utilized at other centers (18–20).

NEC-specific mortality rates were similar between the TC and PT groups, with 13.0 and 23.7% of each group of patients dying, respectively. These findings are well in line previously published mortality rates regarding NEC among both preterm infants (21–23) as well as term-born infants with CHD who develop NEC (6, 9, 24). Interestingly, PT patients were more likely to develop post-NEC complications than their TC counterparts. While no specific complication was more common, 9 PT patients developed bowel obstructions and 4 PT patients developed short bowel syndrome, whereas only one TC patient developed either complication. Although not statistically significant, it remains intuitive that all cases of short-bowel syndrome occurred in our PT group, as NEC seems to more commonly affect the small intestine in preterm patients, as opposed to the colon in CHD patients. Given that we did not have findings of more severe disease among PT patients based on several biochemical markers or Bell's stage, these differences in outcomes show that PT patients may simply represent a more fragile population for which NEC may have more deleterious effects.

In 2019, Bubberman et al. found that TC patients were more likely to have disease of their colon, as opposed to their PT counterparts who were more likely to sustain damage to the small intestine (9). They argued that this discrepancy in site of disease indicates that tissue hypoperfusion is a critical factor in developing NEC among CHD patients, but not for PT patients. No statistically significant difference among site of disease was detected among preterm and CHD patients in our study. However, given that only 3 of the TC patients in our study underwent laparotomy, our analysis was likely underpowered to detect such a difference. Several others studies have shown that term-born infants were diagnosed with NEC much earlier than PT patients (23, 25–27). We did not demonstrate any difference in age at diagnosis in the present study. However, this may be due to the fact that, unlike in those prior reports, there was no difference in the time to initiation of enteral nutrition between term-born and PT patients in our study. Further, those prior studies evaluated all term-born patients who developed NEC, while we specifically focused on term-born patients with CHD.

As has been previously discussed (8, 9), NEC can develop among CHD infants by a variety of mechanisms, including anatomical obstruction of cardiac output, diminished saturation of blood distributed to the lower body or through steal syndrome, in which a substantial portion of the systemic circulation is instead diverted back into the pulmonary circulation. It's clear that all three mechanisms are represented in our TC group, as 91.3% had relatively low oxygen saturation levels at the time of their NEC diagnosis, 78.2% had either a PDA or surgical shunt in place when they developed NEC and 39.1% had congenital left-sided obstructive heart disease.

The current study has several limitations, most notably being its retrospective design and fairly small number of patients to analyze. With only three CHD patients undergoing laparotomy, this left us underpowered to detect certain clinical differences, such as the location of disease. Further, the relatively small sample size could have also left our study underpowered to detect other potential differences in biochemical parameters, imaging results or even specific clinical outcomes. Further, the relatively small sample size prevented performance of a meaningful multivariate analysis. In the future, combining data with additional institutions may be helpful in definitively evaluating whether NEC does indeed develop via different pathophysiological mechanisms in preterm patients vs. term-born patients with CHD. Further, such a study might also be instrumental in determining if different interventions may be applicable to help prevent the development of NEC in these two separate populations.

In conclusion, NEC is frequently treated similarly among both preterm patients and term-born patients with CHD, but there are significant differences among both biochemical parameters and radiological findings among these two groups. This may in fact point toward a difference in the mechanism of disease among these two populations. Further, preterm infants who sustain NEC are more apt to develop post-treatment complications than their CHD counterparts. While these findings may not argue for different treatment strategies, they may suggest a need for different measures to prevent NEC and for how caregivers are counseled for these two very different populations.

## Data Availability Statement

The raw data supporting the conclusions of this article will be made available by the authors, without undue reservation.

## Author Contributions

GF, MR, and BC conceptualized the study, obtained IRB approval, collected study data, performed data analysis, and wrote the manuscript. TL, MP, and HS collected study data, performed data analysis, and made critical revisions to the manuscript. All authors approved submission of the final version of the manuscript.

## Funding

The Department of Surgery at The Mount Sinai Hospital will cover publication fees.

## Conflict of Interest

The authors declare that the research was conducted in the absence of any commercial or financial relationships that could be construed as a potential conflict of interest.

## Publisher's Note

All claims expressed in this article are solely those of the authors and do not necessarily represent those of their affiliated organizations, or those of the publisher, the editors and the reviewers. Any product that may be evaluated in this article, or claim that may be made by its manufacturer, is not guaranteed or endorsed by the publisher.
